# Molecular identification of severe fever with thrombocytopenia syndrome viruses from tick and bitten patient in Southeast China

**DOI:** 10.1186/s12985-020-01391-1

**Published:** 2020-08-05

**Authors:** Yongxi Tong, Qiujing Wang, Yongfeng Fu, Shibo Li, Zhao Zhang, Zheen Zhang, Xuewen Yu

**Affiliations:** 1 Department of Infectious Disease, Zhejiang Province People’s Hospital, Hangzhou, China; 2 Department of Infectious Disease, Zhoushan Hospital, Wenzhou Medical University, Zhoushan, 316021 China; 3 Institute of Biomedical Sciences, Department of Medical Microbiology and Parasitology, School of Basic Medical Sciences, Fudan University, Shanghai, China; 4 McGoven Medical School, 6431 Fannin St., Houston, TX USA

**Keywords:** SFTS, SFTSV, Tick, Transmission

## Abstract

**Background:**

Severe fever and thrombocytopenia bunyavirus (SFTSV) infection causes severe fever and thrombocytopenia syndrome with high mortality. It is extremely rare that a transmitting tick can be directly captured in bite wounds, and that SFTSV can be isolated from both the captured tick and patient’s serum to establish a solid pathogen diagnosis.

**Case presentation:**

We report a case infected with severe fever and thrombocytopenia bunyavirus. The 69-year-old male patient presented with fever and tenderness on two lymph nodes in the right groin. A visible tick bite mark appeared on right upper quadrant of the patient’s abdomen, and a live tick was captured in the bite wound upon physical examination. The virus was detected in both the blood of the patient and in the tick that stayed in the bite wound for 7 days. The phylogenetic analysis indicated that the SFTSV isolated from the tick and the patient’s serum sample belonged to type B, in which the L/S segment of these two isolates shared 100% homology, while the M segment had 99.9% homology. The bitten patient was given various supportive care, but eventually died of multiple organ failure.

**Conclusion:**

The present case provides strong evidence of SFTSV transmission from *H. longicornis* to humans, and suggests that direct cross-species transmission can occur without additional intermediate hosts.

## Background

Severe fever with thrombocytopenia syndrome (SFTS) is an infectious disease that has been reported in 20 provinces in China, including Henan, Hubei, Shandong, Anhui, Liaoning and Zhejiang [1]. Recently, SFTS has also been reported in Japan and Korea [2, 3]. The causative SFTS pathogen is severe fever with thrombocytopenia syndrome virus (SFTSV), which is a member of the Huaiyangshan Banyangvirus species, Banyangvirus Genus, Phenuiviridae family [4]. SFTSV or viral genome fragments were detected in both ticks of *Haemaphysalis longicornis* and *Boophilus microplus*, which can be collected from domestic animals of cattle, goats and dogs [5], and the serum samples obtained from infected animals of sheep, cows and dogs, suggesting that ticks serve as a key vector for SFTSV transmission [6, 7]. Data on the transmission of SFTS from ticks to humans remain uncertain [8], and it was merely in one case that it was found that the SFTSV detected from the tick was identical to that detected in the patient’s blood and CSF [9]. We report a case where SFTSVs were isolated from a tick that lived in the bite wound of a SFTS patient for 6 days, and from the patient with active viral replication.

## Case presentation

The patient was a 69-year-old male plasterer, who resided in Daishan Island in Southeast China. On July 30, 2014, the patient was bitten by a tick on the right upper quadrant of the abdomen. After 2 days, the patient developed a 39.0 °C fever with fatigue and nausea, and was admitted to a local clinic. Leukopenia was noted. The blood specimen collected on admission tested positive for SFTSV using reverse transcription-polymerase chain reaction (RT-PCR) assay [10] at the Centers for Disease Control and Prevention (CDC).

After 5 days of antibiotics and ribavirin treatment, the patient remained feverish, and was transferred to Zhoushan Hospital, which is a regional medical center, on August 6. Upon arrival at Zhoushan Hospital, the patient had a 38.8 °C fever, but was conscious. A visible tick bite mark appeared on right upper quadrant of the patient’s abdomen. The patient complained about the tenderness on two lymph nodes in right groin upon physical examination. A dark red pimple-like bump was located on the lower left chest, and was punctured with a needle. The surviving tick was captured from the bump. The computed tomography (CT) of the patient’s chest revealed calcified pleural nodules on the lower lobe of the right lung and inflammation on the upper lobe of both lungs.

The laboratory tests revealed that the alanine and aspartate aminotransferase levels were elevated to 247 U/L and 987 U/L on day 10, respectively. Furthermore, the lactic dehydrogenase and creatine kinase levels continuously increased to 3211 U/L and 3860 U/L, respectively. However, the creatinine, blood urea nitrogen and serum albumin levels remained normal. The white blood cell count progressively decreased during the course of the patient’s illness. The platelet count was persistently low, which was accompanied by activated partial thromboplastin times (APTTs). The serum viral load, which was determined by qRT-PCR (PrimeScript RT-PCR Kit, Takara Bio Inc., Japan), remained steady at 9.0 × 10^4^ copies/μl until the patient died. The qRT-PCR was performed using primers and probes specific to a conserved region of the SFTSV S segment: Forward primer P3: 5′-ACT CTC TGT GGC AAG ATG CCT TCA-3′; Reverse primer: P4: 5′-AGT TCA CAG CTG CAT GGA GAG GAT-3′, the probe: 5′(FAM)-AAT GTG AAG ATG CGT GGA GCC AGC AA(TAMARA)-3′. The RNA standard was prepared with the S-segment plasmid transcribed by T7 RNA polymerase (Thermo Scientific™, Shanghai, China), according to manufacturer’s instructions. The RNA quantification was carried out using a ND-2000c spectrophotometer (NanoDrop, Wilmington, USA). The standard curves for the SFTSV qRT-PCR were developed with 10^6^, 10^5^, 10^4^, 10^3^, 10^2^ and 10 copies/μL RNA standards.

On the 10th day, the patient suddenly experienced convulsions and obnubilation. The findings from the multi-slice CT scan of the patient’s brain was unremarkable. The patient was transferred to the intensive care unit, and received tracheal intubation with artificial ventilation. The patient died of multiple organ failure on the following day (August 11, 2014).

The viral RNA was isolated from the second blood sample collected on August 6 using the QIAamp MinElute Virus spin kit (QIAGEN, Hilden, Germany), and RT-PCR (PrimeScript RT-PCR Kit, Takara Bio Inc., Japan) was performed. The full length of all three genome segments (L, medium [M] and small [S]) were obtained by overlapping the polymerase chain reaction (PCR) with the primers (see Additional file 1) [7]. The amplified PCR products were directly sequenced (ABI Prism 3100 genetic analyzer; Applied Biosystems, Foster City, CA, USA). The generated sequences were assembled using the DNASTAR 6.0 software. The complete sequences of the L, M and S segments were deposited in GenBank (Accession: KR017844, KR0178863, MT236315, MT236316, MT236317 and MT236318).

The tick that was isolated from the patient was identified as *Haemaphysalis longicornis* by morphology. The tick was kept alive for 6 months in a dry plastic tube, which was placed in an incubator at 25 °C with 90% relative humidity. The tick was molecularly confirmed as *Haemaphysalis longicornis* by sequencing the cytochrome c oxidase 1 gene. The RNA was isolated from the tick using a QIAamp MinElute Virus spin kit (QIAGEN, Hilden, Germany). The RT-PCR assay revealed that the SFTSV was RNA positive. Then, the resultant PCR fragments were sequenced, and the sequences of all three SFTSV genome segments were obtained.

**Fig. 1:**
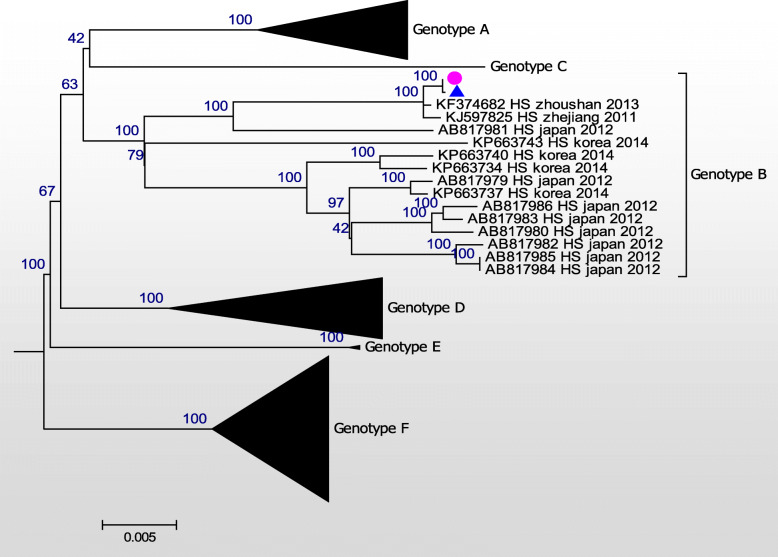
A maximum phylogeny based on the full-length sequences of the L segment of the two isolates obtained from the patient’s serum (pink dot) and the tick (blue triangle), and other representative strains of the SFTSV. The scale bar represents the mean nucleotide substitutions per site

**Fig. 2:**
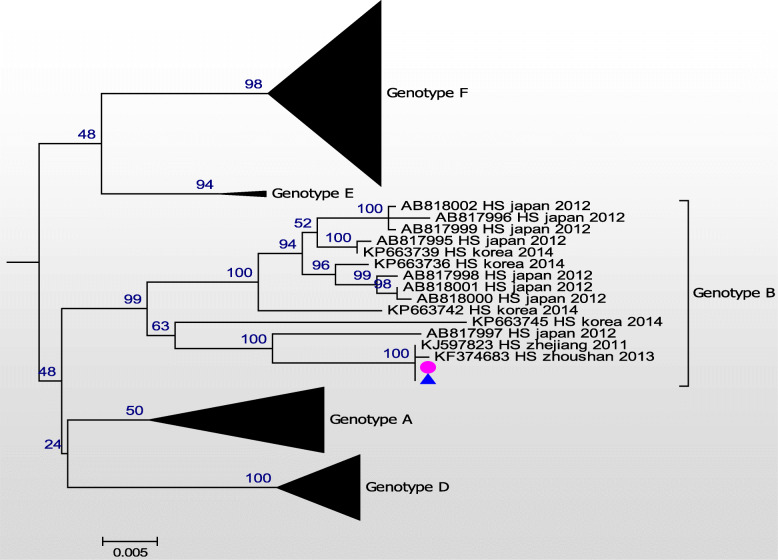
A maximum phylogeny based on full-length sequences of the S segment of the two isolates obtained from the patient’s serum (pink dot) and the tick (blue triangle), and other representative strains of the SFTSV. The scale bar represents the mean nucleotide substitutions per site

**Fig. 3:**
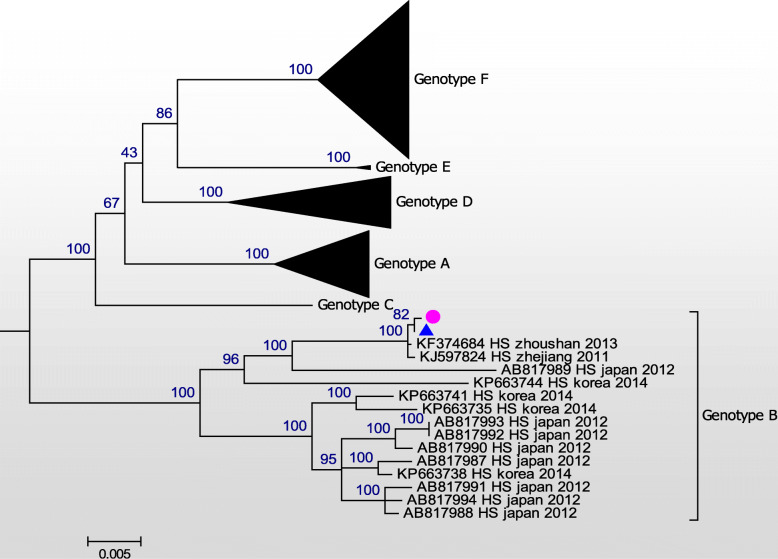
A maximum phylogeny based on full-length sequences of the M segment of the two isolates obtained from the patient’s serum (pink dot) and the tick (blue triangle), and other representative strains of the SFTSV. The scale bar represents the mean nucleotide substitutions per site

Consequently, the whole genomic sequences of these two SFTSV isolates were established. These two isolates shared 100% homology in both the L and S segments (Figs. 1 and 2), and 99.9% homology in the M segment (Fig. 3). A difference from C to T at nt948 and another difference from T to A at nt1713 were detected in the human isolate, when compared to the tick isolate. Phylogenetic analysis was performed using all whole genome sequences from the NCBI database with the MEGA7 software [11]. Maximum likelihood (ML) trees were reconstructed using the bootstrap method, with 100 replications. Six SFTSV genotypes were classified (A-F) [12]. Both isolates in the present study belonged to genotype B, with high bootstrap values.

## Discussion and conclusion

SFTSV has been suggested to be the causative pathogen of SFTS by recent studies [13]. SFTS is an insect or tick-borne contagious viral disease, and *Haemaphysalis longicornis* represents as an intermediate vector in the SFTSV transmission chain [2]. In the present case, the tick was captured alive from the bite lesion of the patient, and was identified as *Haemaphysalis longicornis*, which is consistent with a report [1]. A steady high viral load is an independent risk factor for fatal outcomes of SFTS. The serum kinetic SFTSV load on day 1, day 2 (the tick was captured), and days 3–5 after admission continuously increased. The patient died of disseminated intravascular coagulation. The sustained tick bite provided the extended opportunity to inject viruses into the wound, allowing these to enter the blood stream, and spread to the organs and tissues for replication [14], thereby elevating the serum virus load. Since there is no direct antiviral treatment for SFTS, the physical protection of tick bites represent an important measure to prevent the transmission and infection of SFTSV. A careful physical examination is also important for the diagnosis of SFTS.

Ticks are the intermediate vectors for SFTSV transmission. SFTSVs have been isolated in ticks from China, South Korea and Japan. High homology in viral gene sequences has been reported among isolates obtained from ticks and anthropogenic infectious disease isolates. What is unique in the present case was that the tick was directly captured alive from the wound of the patient. Surprisingly, the tick remained attached to the wound for at least 7 days. The tick was housed for six more months until testing the SFTSV genes. All sequences, except for the two bases in the two isolates, were identical, and belonged to type B, providing genetic evidence that the SFTSV infection in the present case was likely transmitted by the tick. The difference of the two nucleotides in the M segment, between the tick and patient isolates, could be caused by sequencing errors or replication-induced mutations [12]. The evidence that the tick SFTSV was likely transmitted to humans, without additional intermediate hosts, highlights the transmission efficiency of SFTSV, and may cause an outbreak in regions where ticks are rampant.

## Supplementary information

**Additional file 1.** Specific primers used to amplify the whole genome segments (L, M and S).

## Data Availability

Not applicable.

## Abbreviations

SFTS: Severe fever with thrombocytopenia syndrome
SFTSV: Severe fever with thrombocytopenia syndrome bunyavirus
APTTs: Activated partial thromboplastin clotting time
RT-PCR: Reverse transcription PCR
CT: Computed tomography
CDC: Centers for Disease Control and Prevention
