# Supplementary material for: Molecular identification of severe fever with thrombocytopenia syndrome viruses from tick and bitten patient in Southeast China
Source: Virol J. 2020 Aug 5;17:122. doi: 10.1186/s12985-020-01391-1 (PMC7409506; doi:10.1186/s12985-020-01391-1)
Supplement: Supplementary file 1 — Additional file 1. Specific primers used to amplify the whole genome segments (L, M and S). [file 12985_2020_1391_MOESM1_ESM.docx]

Table 1. Specific primers used to amplify the whole genome

| Primer | Primer sequence (5’-3’) | Base position | Amplicon size(bp) |
| --- | --- | --- | --- |
| **L segment** |  |  |  |
| 5-Ter-F1 | ACACAAAGACGCCCAGATGA | 1-20 | 1132 |
| L1112-R1 | GACATCTAGAAGGCACTTTGC | 1132-1112 |  |
| 5-Ter-F2 | ACGCCCAGATGAACTTGGAA | 9-28 | 1062 |
| PT1050-R2 | AGCCTGAGTCGGTCTTGATGT | 1070-1050 |  |
| L968-F1 | AGGAGCAACAAGCAAACATCAT | 968-989 | 1305 |
| L2253-R1 | CAATGTACCAGCTGTTCACCA | 2273-2253 |  |
| P991-F2 | GAATCGAGGGACAGTCAAAC | 991-1010 | 1250 |
| P2221-R2 | TTGTGGACCGGCCTGAGATG | 2240-2221 |  |
| L2136-F1 | TGGATTGCATGGTGCGAATTG | 2136-2156 | 1020 |
| L3135-R1 | GATCAGATGACCTAGACTCAG | 3155-3135 |  |
| Seq2F2 | GAACATTCCATGCCATCTCAG | 2208-2228 | 914 |
| Seq1R2 | GTGAGCTAAAAACCTTAGGTC | 3121-3101 |  |
| L3031-F1 | CATGCCAGCYAAATTCCACAG | 3031-3051 | 1125 |
| L4135-R1 | TTCTTGTTGGCAGCTCTCCTG | 4155-4135 |  |
| L3058-F2 | TTGGGCTGCCATTTCCATGTT | 3058-3078 | 1073 |
| P4111-R2 | GGACTCCAGGATTCTCATCT | 4130-4111 |  |
| PN4010-F1 | GGAACTCTCAGCCACTCTGTT | 4010-4030 | 974 |
| L4963-R1 | GTAGAGAAGGCCTCTATGATC | 4983-4963 |  |
| PN407l-F2 | TGAACAGGATGGGCCTTCCTG | 4071-4091 | 884 |
| L4934-R2 | CTTGTACTCCTCAGTGTATGG | 4954-4934 |  |
| L4843-F1 | TAGCCTAGAAGCTGAGAAGAG | 4843-4863 | 900 |
| P5722-Rl | CCTTGGGTCTTCCTATCATTT | 5742-5722 |  |
| P489l—F2 | TATCTCCATCCTCAAGCATGT | 4891-4911 | 829 |
| P5699-R2 | AGGAGAACTGAGGCATGTGA | 5719-5699 |  |
| L5577-F1 | ACACTGATATATCAGAGTCAGC | 5577-5598 | 792 |
| 3-Ter-R1 | ACACAAAGACCGCCCAGATC | 6368-6349 |  |
| P5617一F2 | CAATCGAGACCTCTTCTCCT | 5617-5636 | 744 |
| 3-Ter-R2 | ACCGCCCAGATCTTAAGGAA | 6360-6341 |  |
| **M segment** |  |  |  |
| M—Fl | ACACAAAGACGGCCAACAATG | 1-21 | 630 |
| M611-Rl | GTCTGGGAATTCACTTTGGC | 630-61l |  |
| M-F2 | ACGGCCAACAATGATGAAAGTC | 9-30 | 596 |
| M585-R2 | TCTTCAGCTCCAGAAATGTC | 604-585 |  |
| M526一F1 | GATAGGGTTCTCTGGATAGG | 526-545 | 455 |
| M960-Rl | ATCCCTCCATATGACACAACG | 980-960 |  |
| M543一F2 | AGGTGATGTTGCTTGTCAGC | 543-562 | 402 |
| M924-R2 | TGCACAAGTGAGCATCTACAC | 944-924 |  |
| M810-Fl | GTRCAAGAGAGCTCATCCAAG | 760-780 | 614 |
| M1354-Rl | TTCTTTGCAGGGTAGCACTG | 1373-1354 |  |
| M838-F2 | TGTGCTAYAAGGAAGGGACTG | 788-808 | 565 |
| M1331一R2 | GGATTTTTTAGAAACTCACGAC | 1352-1331 |  |
| M1267-F1 | GAATTCACATTTGAGGGTAGTT | 1267-1288 | 678 |
| M1922-Rl | ACCGGGCATCAGGAACAAAAT | 1942-1922 |  |
| M1285-F2 | AGTTGCATGTTCCCAGATGG | 1285-1304 | 589 |
| M1853一R2 | TGAGACATTTTGAGTCYGGAC | 1873-1853 |  |
| M1705-F1 | TGTGATGAGATGGTCCATGCT | 1705-1725 | 626 |
| M2311-Rl | ACCTCCATATCTGAGCCCAA | 2330-2311 |  |
| M1751-F2 | AAGGGAGCGGAAATATGAAGG | 1751-1771 | 553 |
| M2284-R2 | ACACTCACACCCTTGAAGAC | 2303-2284 |  |
| M2220-Fl | AGAGCTAACAATGCCCTCAG | 2220-2239 | 581 |
| M2780-Rl | AGACTTTGGCGCCTGTCATGC | 2800-2780 |  |
| M2247-F2 | GAGGACATTCCACCCCATGA | 2247-2266 | 516 |
| M2742-R2 | ACATTTGTCACYTCCCCTGTG | 2762-2742 |  |
| M2655-F1 | CCCCCTGGACATCACAGCTAT | 2655-2675 | 460 |
| M3095-Rl | CCCATTGCCAAACAAGGCAT | 3114-3095 |  |
| M2690-F2 | CTGTYAATTATAGAGGCCTTCG | 2691-2711 | 387 |
| M3058-R2 | CTTGGGATATTTGCCCCTGT | 3077-3058 |  |
| M2943一F1 | ACTGAACTGTGGGGGACATG | 2943-2962 | 435 |
| M3358-Rl | ACACAAAGACCGGCCAACACT | 3378-3358 |  |
| M3014一F2 | TTGTGGATGGCAGCTACATG | 3014-3033 | 351 |
| M3343-R2 | AACACTTCAAYRGAACCTCCAT | 3365-3344 |  |
| **S segment** |  |  |  |
| S1-Fl | ACACAAAGAACCCCCAAAAAAGG | 1-23 | 711 |
| S692-R1 | CCCTTGGCCTTCAGCCACTT | 711-692 |  |
| S9-F2 | AACCCCCAAAAAAGGAAAGACG | 9-30 | 661 |
| S650-R2 | AAGACAGAGTTCACAGCAGC | 669-650 |  |
| S562-F1 | GCAAGATGCCTTCACCAAGA | 562-581 | 462 |
| S1003-Rl | ACAGTGTCTTGGATGAGGATG | 1023-1003 |  |
| S603一F2 | GAGCCAGCAAGACAGAAGTT | 603-622 | 374 |
| S956-R2 | CCAYATCTGATGGCACACTAT | 976-956 |  |
| S829-Fl | GACAAAATTAGACCTCCTTCG | 829-849 | 643 |
| S1452-Rl | TGTACTACAAGGACATGAGG | 1471-1452 |  |
| S857-F2 | ACCAATGGCTGGCCAATCTCT | 857-877 | 494 |
| S1341-R2 | TCAAGAACAGCTGGGCAATG | 1350-1341 |  |
| S1259一Fl | TATCATGTCCCCTTCAAAAAG | 1259-1279 | 488 |
| S1725-Rl | ACACAAAGAACCCCCTTCATTT | 1746-1725 |  |
| S1313-F2 | GGCCCCGCCAGTTCTCTCT | 1313-1331 | 426 |
| S1717-R2 | AACCCCCTTCATTTGGAAACCA | 1738-1717 |  |
